# Subacute Hydrocephalus Revealing a Cryptococcus Infection in a Seven-Year-Old Child

**DOI:** 10.7759/cureus.56372

**Published:** 2024-03-18

**Authors:** Tarik Belokda, Hajar Hamadi, Yassine Ait M'Barek, Lamia Benantar, Khalid Aniba

**Affiliations:** 1 Neurological Surgery, Ibn Tofail Hospital, Mohammed VI International University Hospital, Marrakech, MAR; 2 Neurological Surgery, Mohammed VI International University Hospital, Marrakech, MAR

**Keywords:** pediatric hydrocephalus, intracranial hypertension, hiv, ventriculoperitoneal shunt, cryptococcus neoformans, cryptococcal meningitis

## Abstract

Cryptococcal meningitis (CM) is the third most common neurological complication in immunocompromised patients and is usually associated with high rates of morbidity and mortality. The most common complication of CM is intracranial hypertension (ICH), and it constitutes a poor prognosis factor. This case report describes a case of subacute onset hydrocephalus revealing a human immunodeficiency virus (HIV)-associated CM in a seven-year-old girl requiring cerebrospinal fluid diversion and fungal treatment with a favorable outcome.

## Introduction

Cryptococcal meningitis (CM) due to Cryptococcus neoformans is the third most common neurological complication in human immunodeficiency virus (HIV)-positive patients and is usually associated with high rates of morbidity and mortality [1,2]. The overall mortality rate is approximately 500,000 patients per year worldwide, with severe morbidity in 95% of cases in developed countries [3]. The most common complication of CM is intracranial hypertension (ICH), and it constitutes the most accurate poor prognosis factor in HIV-positive patients, leading to impaired mental status, neurological deterioration, and severe disability [1-3].

Several therapeutic options can be used to treat ICH in cases of HIV-associated CM, including antifungal drugs, serial lumbar puncture (LP), or shunt treatment [1,4]. The risk of shunt infection or obstruction as well as peritoneal Cryptococcus seeding from direct transport of the infection has historically discouraged surgeons from implanting cerebrospinal fluid (CSF) shunts in patients with HIV and CM [1]. However, shunt placement should be performed within an appropriate timeframe to manage ICH symptoms and prevent further complications [2]. We report a case of ICH secondary to CM in a seven-year-old girl, revealing an HIV infection.

## Case presentation

The patient is a seven-year-old girl admitted to the pediatric emergency department for symptoms of increased intracranial pressure (ICP), including holocephalic headaches and vomiting followed by decreased visual acuity as well as an isolated episode of generalized tonic-clonic seizure one day before her admission. The patient's medical history included undocumented seizures at the age of three months, purulent otitis media, and infected bullous dermatitis at the age of six years for which the patient was treated with antibiotics with no further follow-up or investigations (the patient's parents did not have any documents related to her history, and no further doctor consults were made since the parents did not seek further treatment). The patient had no siblings, and the family history was unremarkable (no prior history of HIV). Clinical examination showed a conscious, afebrile girl with a Glasgow Coma Scale (GCS) of 15. She exhibited macrocrania, measuring at +2 standard deviations, with esotropia in the left eye. Fundoscopic examination showed stage 2 bilateral papilledema. The rest of the physical examination was normal. The patient underwent a basic blood panel, which was within the normal range.

**Figure 1:**
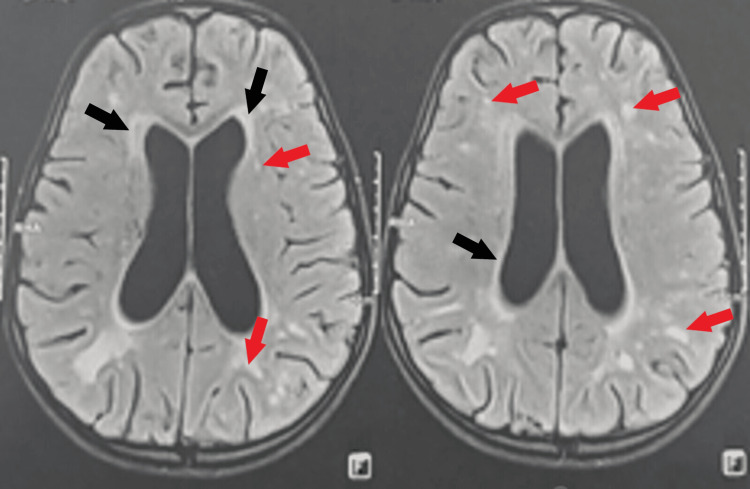
Preoperative MRI of the brain. Brain MRI in axial planes FLAIR sequence revealed moderately dilated lateral ventricles with a periventricular hypersignal, indicating its active characteristic (black arrows). Also visible are multiple lesions in hypersignal FLAIR (red arrows) located in the periventricular white substance and the right internal capsule, indicating ischemia probably due to acute demyelinating encephalomyelitis. FLAIR, fluid-attenuated inversion recovery; MRI, magnetic resonance imaging

A magnetic resonance imaging (MRI) of the brain confirmed the presence of a moderate active triventricular hydrocephalus (Figure 1).

Following the imaging results and after all contraindications were ruled out, a lumbar punction with CSF analysis was performed. The results of the fluid analysis showed a glucose level of 0.52 g/L (normal = 0.6 g/L), a protein level of 0.56 g/L (normal < 0.4 g/L), and the presence of Cryptococcus neoformans yeast. Once the patient was diagnosed with CM, an HIV test was carried out (with parental consent), and the results returned positive. Further tests were carried out, including a CD4 count, which was extremely low at 4/mm³ (normal range 500-1,000/mm³), and a viral load, which was 40 copies/mL, implying a low viral load, signifying that the virus was likely not actively multiplying at the time. These test results were consistent with severe immunodeficiency.

The patient was hospitalized at our Department of Neurosurgery at Ibn Tofail Hospital, Mohammed VI International University Hospital in Marrakech for the management of her ICH. Once the diagnosis and therapeutic management were explained to the parent, consent was obtained for the placement of a ventriculoperitoneal (VP) shunt. After the surgical procedure was completed, the patient's symptoms improved, and she was transferred to the infectious disease department for further management of her HIV and cryptococcal infections. She was immediately started on a course of antiretroviral therapy associated with antifungal treatment using intravenous Amphotericin B at a dose of 0.7 to 1 mg/kg/day for two weeks associated with oral 5-flucytosine at a dose of 100 mg/kg/day (divided into four doses a day) for two weeks, which was then followed with oral Fluconazole at a dose of 200 mg/day for six months.

Patient follow-up at 15 days post-op showed the total disappearance of her intracerebral hemorrhage (ICH) symptoms. Follow-up at 1 month, 3 months, and 6 months showed no signs of recurring ICH and no complications related to the VP shunt, as well as normalized CD4 count and decreased viral load. A regular follow-up of the patient's CD4 count and viral load is mandatory to evaluate the patient's immunodeficiency status and adjust her ongoing treatment to prevent opportunistic infections and complications. An HIV test was proposed to the mother but consent was not obtained.

## Discussion

CM is a common opportunistic infection in immunocompromised hosts and the leading cause of meningoencephalitis in sub-Saharan Africa and Southeast Asia [5,6]. It typically affects the respiratory tract and then disseminates hematogenously to the brain [6]. To date, even with effective pathogen-directed therapy and the widespread use of highly active antiretroviral therapy (HAART), CM is still a refractory disease with a long course and poor prognosis [5,7]. Hydrocephalus is a well-described common complication of CM [2,8]. The presence of ICH has been reported in over half of the cases of CM and is associated with higher rates of mortality and morbidity [2,8,9]. The most common clinical manifestations include headache, nausea, fever, visual impairment, cranial nerve damage, and loss of consciousness, which can all be attributed to the development of hydrocephalus [8,10]. Our patient clinical presentation is consistent with ICH manifestations likely due to her triventricular hydrocephalus.

Management of elevated ICP is of paramount importance and usually involves serial lumbar punctures or conventional dehydration in internal medicine [1,7,9]. In refractory cases with uncontrollable ICH and with the presence of concomitant hydrocephalus, surgical indications for VP shunting have been proposed if repeated lumbar punctions are insufficient to improve the symptoms [7]. While the choice of treatment and the timing are subject to controversy, many authors recommend that the shunt placement should be performed within an appropriate timeframe to avoid long-term complications from CM [2,8]. However, some cases of hydrocephalus secondary to CM do not improve after CSF diversion through a VP shunt, which suggests that selecting the right patients for this procedure is important [6,8]. The selection criteria for VP shunting are not well established, but several factors contributing to this decision are reported in the literature. These include extremely high ICP with or without hydrocephalus, increased opening pressure with severe clinical signs of ICH, a favorable response to large volume removal of CSF via lumbar puncture but requiring continuous lumbar CSF drainage to remain neurologically asymptomatic, the presence of papilledema with potential vision loss, symptom recurrence despite maximal antifungal therapy, and radiological findings such as meningeal enhancement, single or multiple nodules, cerebral edema, or hydrocephalus [4,9-11]. The timing of the surgery according to Tang is dependent on the patient’s symptoms, as the presence of acute signs and symptoms of hydrocephalus are factors predictive of a better outcome after shunt placement [6,12]. On the other hand, some authors suggest prophylactic shunt placement to avoid irreversible neurological complications [8,11]. In our case, the patient presented with symptoms of high ICP as well as visual impairment and papilledema. In light of these findings and in addition to the presence of hydrocephalus on brain MRI, we preferred VP shunting with no delay to avoid further deterioration of the patient’s vision and prevent these irreversible neurological complications.

Some complications associated with VP shunting include infection (fungal infection or other secondary infections), shunt obstruction, and excessive shunting [7]. However, the benefits may outweigh the risks when compared with the poor neurological prognosis and possible death associated with untreated ICH and hydrocephalus in CM of the immunocompromised [11,13]. Data on long-term outcomes after VP shunting are not easily found since most studies are retrospective and life expectancy in these patients is short, not allowing for a proper follow-up [11,13]. The study carried out by Wen et al. on 72 patients was the first to obtain results of long-term follow-up, showing an improved overall survival rate even in critically ill patients undergoing VP shunting compared with the group of patients who did not [11]. Wen et al. concluded that VP shunting should be applied to avoid death for patients with CM with uncontrollable ICH and rapid development of clinical symptoms, which aligns with our course of treatment [11].

## Conclusions

In summary, patients with AIDS presenting with increased ICH and hydrocephalus secondary to CM could benefit from VP shunt placement. The latter is considered despite potential complications. It is even paramount to shunt patients with rapid onset of ICH symptoms to prevent the unavoidable complications and high risk of mortality if left untreated. The case we present illustrates a better outcome of the indication of the right course of treatment at the optimal time, which shows the importance of a thorough selection of patients undergoing VP shunting for the management of ICH secondary to HIV-associated CM.
